# Linear IgA Bullous Dermatosis in Korea Using the Nationwide Health Insurance Database

**DOI:** 10.3390/jcm13041159

**Published:** 2024-02-19

**Authors:** Yu Rim Kim, Ji Hyeon Kim, Sang Won Kim, Jae Min Lee, Jacob S. Bae

**Affiliations:** 1 Department of Medicine, College of Medicine, Yeungnam University, Daegu 42415, Republic of Korea; 2 Medical Research Center, College of Medicine, Yeungnam University, Daegu 42415, Republic of Korea; kimsw3767@ynu.ac.kr; 3 Department of Pediatrics, College of Medicine, Yeungnam University, Daegu 42415, Republic of Korea; 4 Department of Internal Medicine, College of Medicine, Yeungnam University, Daegu 42415, Republic of Korea

**Keywords:** linear immunoglobulin A bullous dermatosis, Korea, epidemiology, antibiotics

## Abstract

(1) Background: Linear immunoglobulin A bullous dermatosis (LABD) is a rare autoimmune, subepidermal blistering disease, characterized by linear IgA deposits along the epidermal basement membrane. LABD is idiopathic and is associated with medication and systemic autoimmune diseases. (2) Methods: We investigated the demographic characteristics, disease course, causative agents, and associated diseases in Korean patients with LABD. The Korean Health Insurance Review and Assessment Service database was used to obtain data. We identified 670 LABD cases between 2010 and 2022. (3) Results: The annual incidence of LABD was 1.3 per 100,000 persons, with a higher prevalence in individuals ≥60 years old. The patients were treated with dapsone for 30.7 ± 56.7 days, had 1.3 ± 0.7 hospital visits, and were hospitalized for 19.8 ± 19.7 days. Risk factors, including malignancy, commonly preceded LABD. Antibiotic use, specifically vancomycin and third-generation cephalosporins, was a risk factor. The mean age of LABD diagnosis was 55.9 ± 21.7 years. (4) Conclusion: This is the first published study to assess a nationwide cohort for LABD. The incidence of LABD was higher than that in other studies. Most case reports have linked LABD with the administration of specific antibiotics; however, this study shows there were more associations with other conditions.

## 1. Introduction

Linear immunoglobulin A bullous dermatosis (LABD), also known as linear IgA disease (LAD), is a rare autoimmune subepidermal blistering disease with an incidence of 0.2–2.3 cases per million population per year [1], occurring in both children and adults [2]. LABD is characterized by the linear deposition of IgA along the epidermal basement membrane zone. The major, but not exclusive, target antigens are the 97 and 120-kDa proteolytic fragments of BP180 [3,4]. Polycyclic clusters of bullae with central crusting, called strings of pearls, are the most characteristic features, especially in children; however, in adults, their clinical presentation is often polymorphic [5,6]. LABD was first described by Chorzelski et al., who distinguished it from dermatitis herpetiformis [7]. 

LABD is idiopathic. It has been reported to be associated with drug ingestion and systemic autoimmune diseases such as systemic lupus erythematosus (SLE) and ulcerative colitis (UC) [8,9]. Dapsone is the first-line therapy for spontaneous LABD [10], followed by sulfapyridine, steroids, or immunosuppressants in the event of failure or intolerance [11,12,13].

However, information regarding LABD in Korea is lacking because of its rarity and the lack of a well-defined diagnosis in the Korean Classification of Diseases (KCD) or International Classification of Diseases (ICD). Therefore, we conducted a retrospective study based on the current definitions of LABD. In addition, this is the first nationwide study on LABD in Korea. This study aimed to investigate the characteristics of Korean patients with LABD in terms of demographics, disease course, causative agents, and diseases, using the Health Insurance Review and Assessment (HIRA) open-access big data platform.

## 2. Materials and Methods

### 2.1. Data Sources

The National Health Insurance System operated by the Ministry for Health, Welfare, and Family Affairs in Korea provides universal coverage and encompasses approximately 98% of the Korean population. The Korean HIRA database, which comprises data on more than 46 million patients annually (90% of the total population in Korea), includes information from nearly 80,000 healthcare service providers, as of 2011. This comprehensive database, which contains details of patient diagnoses, treatments, procedures, and prescription drugs, serves as a valuable resource for healthcare services research [14,15,16].

### 2.2. Case Definition

**Figure 1:**
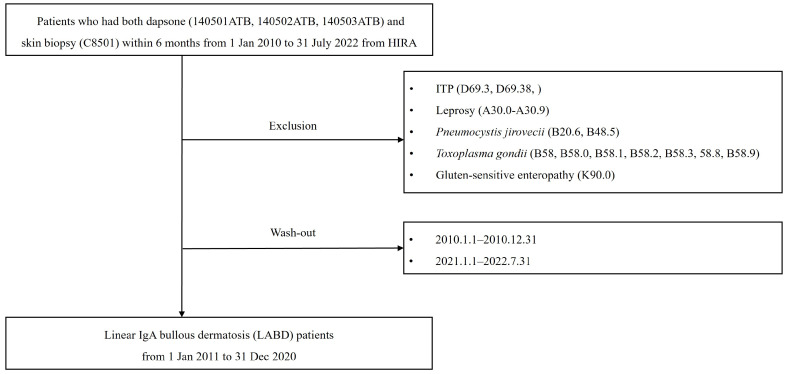
Flowchart illustrating patient selection.

We identified patients diagnosed with LABD from 1 January 2010 to 31 July 2022, from the HIRA database (Figure 1). Given that the KCD does not have diagnostic codes for LABD, we selected patients based on the following criteria: patients who had undergone both dapsone treatment and skin biopsies within 6 months (according to drug and procedure codes). Patients were excluded who were diagnosed with idiopathic thrombocytopenic purpura (ITP), leprosy, *Pneumocystis jirovecii*, *Toxoplasma gondii*, or gluten-sensitive enteropathy for which treatment with dapsone was considered (using diagnostic codes based on the KCD 8th revision, which is a modified version of the ICD). We excluded periods from 1 January 2010 to 31 December 2010 and from 1 January 2021 to 31 July 2022. The reason for excluding patients in 2010 was that patients diagnosed before 2010 may have been considered as new patients in 2010. The reason for excluding patients in 2021–2022 was that numbers of patients may have been underestimated owing to the delay in claiming data. The drug codes used for dapsone were 140501ATB, 140502ATB, and 140503ATB. The procedure codes for skin biopsy were C8501, C8501A00, and C8501B00. The diagnostic codes for ITP included D69.3 (idiopathic thrombocytopenic purpura), D69.38 (other idiopathic thrombocytopenic purpura), and D69.6 (thrombocytopenia, unspecified). The codes for leprosy included A30 (leprosy [Hansen’s disease]), A30.0 (indeterminate leprosy), A30.1 (tuberculoid leprosy), A30.2 (borderline tuberculoid leprosy), A30.3 (borderline leprosy), A30.4 (borderline lepromatous leprosy), A30.5 (lepromatous leprosy), A30.8 (other forms of leprosy), and A30.9 (leprosy, unspecified). The codes for *P. jirovecii* included B20.6 (HIV disease resulting in *P. jirovecii* pneumonia) and B48.5 (pneumocystosis). The codes for *T. gondii* included B58 (toxoplasmosis), B58.0 (toxoplasma oculopathy), B58.1 (toxoplasma hepatitis), B58.2 (toxoplasma meningoencephalitis), B58.3 (pulmonary toxoplasmosis), B58.8 (toxoplasma with other organ involvement), and B58.9 (toxoplasmosis, unspecified). The codes for gluten-sensitive enteropathy included K90.0 (coeliac disease).

### 2.3. Statistical Analysis

The age-standardized incidence rate of LABD was calculated adjusting for the annual incidence (per 100,000 individuals) in the mid-year 2016 population. This adjustment involved multiplying the number of LABD cases as the numerator and the annual Korean population based on the HIRA database as the denominator by the correspondingly aged population in 2016. Data are presented as the mean ± standard deviation.

## 3. Results

### 3.1. Patient Characteristics

During the 10-year study period, 670 patients were enrolled. The annual incidence was calculated to be 1.3 per 100,000 individuals (Table 1). Of these, 46 (6.9%) patients were aged 0–19 years of age, 105 (15.7%) were aged 20–39 years, 199 (29.7%) were aged 40–59 years, and 320 (47.8%) were aged 60 years and older. The average age of the subjects was 55.9 ± 21.7 years. The study included 394 male (46.2%) and 458 female (53.8%) patients. The annual incidence of LABD was the highest in the ≥60-years age group. LABD incidence rate in individuals ≥60 years was 3.2/100,000 persons–year, which was 6.4-fold higher than that in 0–19-year age group. Patients with LABD received dapsone for 30.7 ± 56.7 days, had 1.3 ± 0.7 hospital visits, and were hospitalized for 19.8 ± 19.7 days.

### 3.2. Trend Analysis of Linear IgA Bullous Dermatosis

**Figure 2:**
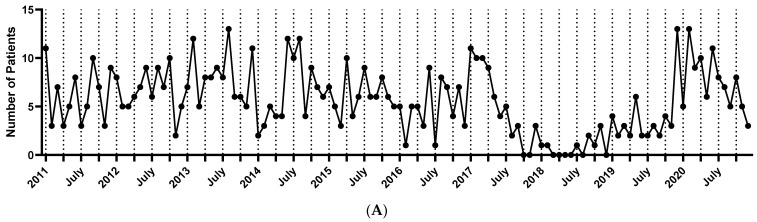

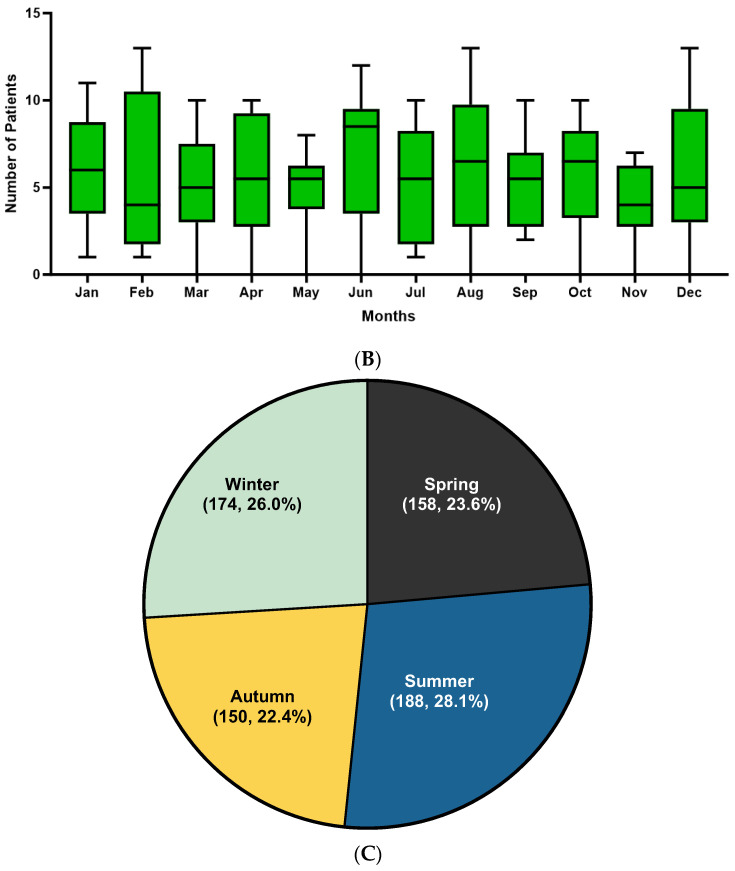
(**A**) Monthly incidence of linear IgA bullous dermatosis during study period. (**B**) Monthly trend of linear IgA bullous dermatosis. (**C**) Seasonal trend of linear IgA bullous dermatosis.

Figure 2A shows the monthly incidence of LABD between 2011 and 2020. The period from 2017 to 2019 showed a lower incidence of LABD than other periods, and there was a shortage of dapsone in Korea during the same period. The peak incidence rate occurred in August 2013. Between 2011 and 2020, June displayed the highest number of monthly cases, totaling 70 (average 7.0), whereas November had the lowest number at 41 (average 4.1) (Figure 2B).

Summer had the highest patient count (28.1%), with June being the peak. LABD diagnoses were most common in summer (28.1%), followed by winter (26%), spring (23.6%), and autumn (22.4%) (Figure 2C).

**Figure 3:**
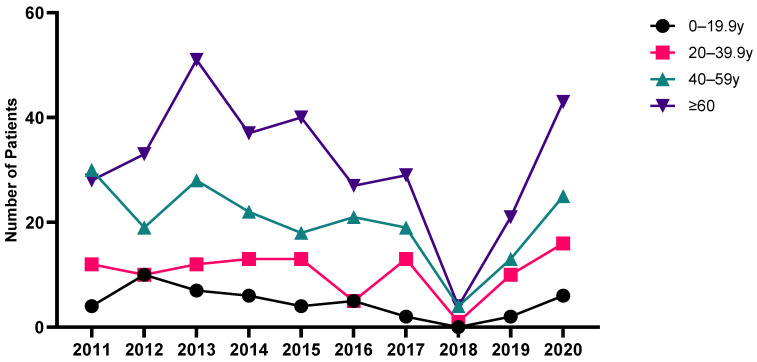
Annual trend analysis of linear IgA bullous dermatosis by age group.

Figure 3 shows the annual incidence trends according to age group. Comparing age groups, those aged 60 years and older consistently accounted for a significant and increasing portion of the patient population. When comparing age groups, the incidence was the highest in the ≥60-years age group, and the incidence was higher in the older group.

### 3.3. Associated Risk Factor of Linear IgA Bullous Dermatosis

**Figure 4:**
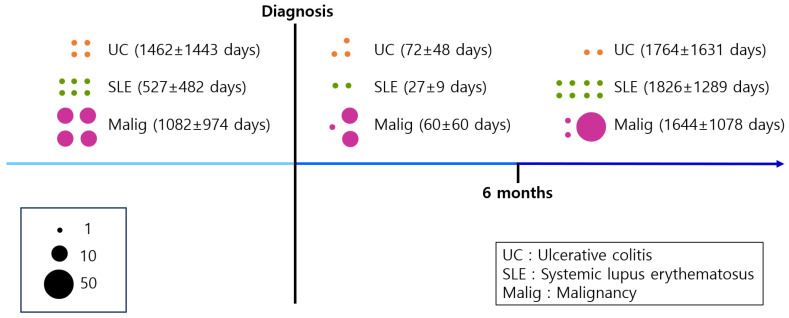
Systemic disease associated with LABD.

Common conditions preceding LABD diagnosis included UC, SLE, and malignancy, with malignancy being the most common (Table 2). Figure 4 shows the timeline of the systemic diseases associated with LABD. A total of 113 patients (16.9%) were diagnosed with malignancy before or after LABD diagnosis. In total, 40 patients were diagnosed with malignancy before the diagnosis of LABD, while 21 patients and 52 patients were diagnosed with malignancy within and after 6 months following the diagnosis of LABD, respectively. The mean time from malignancy diagnosis to LABD diagnosis was 1082 ± 974 days, and the time from LABD diagnosis to malignancy diagnosis was 60 ± 60 days in patients within 6 months and 1644 ± 1078 days in patients after 6 months. Sixteen patients (2.3%) were diagnosed with SLE before or after LABD diagnosis. Six patients were diagnosed with SLE before the diagnosis of LABD, and two and eight patients were diagnosed with SLE within and after 6 months of LABD diagnosis, respectively. The time from SLE diagnosis to LABD diagnosis was 527 ± 482 days, and the time from LABD diagnosis to SLE diagnosis was 27 ± 9 days in patients within 6 months and 1826 ± 1289 days in patients after 6 months. Nine patients (1.3%) were diagnosed with UC before or after LABD diagnosis. Four patients were diagnosed with UC before the diagnosis of LABD, and three and two patients were diagnosed with UC within and after 6 months of LABD diagnosis, respectively. The average time from UC diagnosis to LABD diagnosis was 1462 ± 1443 days, and the average time from LABD diagnosis to UC diagnosis was 72 ± 48 and 1764 ± 1631 days in patients within and after 6 months, respectively.

**Figure 5:**
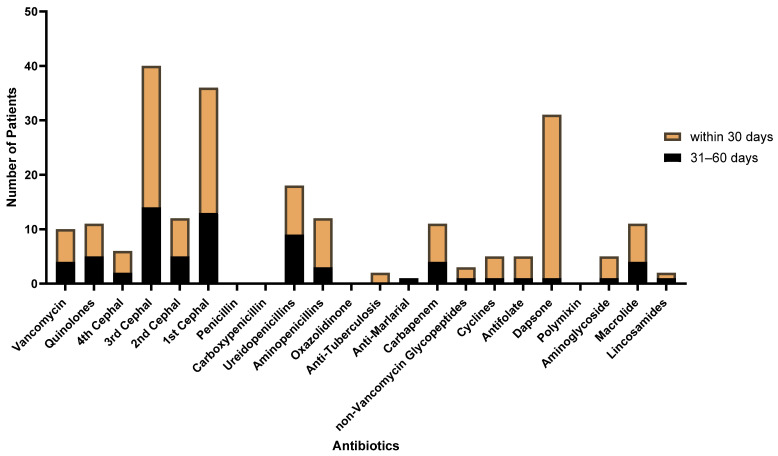
Frequency of antibiotic use concurrent with LABD biopsy.

With respect to vancomycin, which is known to be a risk factor, four people used vancomycin between 31 and 60 days before diagnosis, and six people used it within 30 days before diagnosis (Figure 5, Table S1). The cephalosporin class of antibiotics was used more frequently than were other antibiotics, with 40 third-generation and 36 first-generation cephalosporins.

## 4. Discussion

In this study, we examined the epidemiological characteristics of patients with LABD in Korea over the past 10 years and the associated medications and systemic diseases. 

Lings et al. reported an estimated incidence of LABD of 0.67 per million per year in Denmark, and the mean ages at disease onset in children and adults were 2.7 and 56.8 years, respectively [1]. During 1985–2017, Garel et al. investigated 69 patients based on the French institutional pharmacovigilance database [17]. The male-to-female ratio was 1.3 and the median age was 57 years (range 3–97). In an Italian study group, Genovese et al. reported an average age at diagnosis of 45.7 years (range 0.9–93 years) and bimodal age with a mean age of 5.4 years in the child group <16-year-old and 60.6 years in the adult group. The overall male-to-female ratio was 1.2 [18]. Horiguchi et al. reviewed 213 patients with LABD by summarizing papers and conference abstracts reported between 1975 and 2006 in Japan and classified LABD into an infantile type, aged 15 years or younger, and an adult type, aged 16 years or older [6]. They reported peak incidence between 0–5 years old and 61–75 years old and an increase in IgA/IgG type with age.

In our study, the annual incidence was 1.3/100,000 people. Mean age of diagnosis was 55.9 ± 21.2 years. The ≤19-years age group had the lowest rate (6.9%), whereas the 60 and over age group had the highest rate (47.8%). In our study, unlike other studies [6,18], we did not observe a bimodal age distribution, which may be due to differences in case definition and patient selection, which may have excluded children, those diagnosed without biopsy, or those treated with steroids alone. 

In our study, LABD occurred most frequently in summer. There has been little research on the seasonality of LABD, and it is difficult to accurately determine the cause.

One hypothesis is that sunlight is a common trigger factor for autoimmune skin manifestations [19]. Low doses of ultra-violet B (UVB) have anti-inflammatory effects. However, high doses of UVB cause cell apoptosis, and higher doses of UVB cause cell necrosis and worsen the autoimmune reaction [20]. 

Systemic agents such as dapsone, sulfonamides, and corticosteroids have demonstrated efficacy in the treatment of LABD. Dapsone is the recommended first-line therapy either as monotherapy or combination therapy [10]. Alternatives include sulfonamide drugs such as sulfapyridine, sulfamethoxypyridazine [12], and colchicine [21]. In refractory cases, corticosteroids, immunosuppressive agents, or antimicrobials, such as oxacillin and erythromycin, may be considered. Newer strategies such as intravenous immunoglobulin and immunoadsorption [11] show promise for unresponsive cases or those with side effects [13]. Recent therapeutic trends involve the use of rituximab [22] and anti-tumor necrosis factor (TNF)-α inhibitors for refractory cases [23,24]. Furthermore, one patient with LABD and chronic idiopathic urticaria reported the potential benefits of omalizumab [25]. 

In drug-induced LABD, prolonged immunosuppression is generally unnecessary, and spontaneous resolution often follows the cessation of the causative agent [24]. Fortuna et al. reported that despite discontinuation, up to 50% of patients may require additional therapy [26]. This is crucial for preventing disease amplification triggered by immunological signals, leading to a self-maintaining immune response.

Genovese et al. reported a median dapsone treatment duration of 26.2 months [18]. Lings et al. demonstrated that the mean treatment duration (dapsone in combination with prednisone) in the idiopathic group was 6.9 years (range 1–22 years, median 3 years). Mean duration of treatment in the adult LABD group overall was 4.1 years (range 1 month–22 years, median 2 years) [1]. 

Our study found that patients with LABD received dapsone for an average of 30.7 ± 56.7 days. In contrast to previous studies that examined the combination of dapsone and prednisone, our study focused solely on dapsone, which resulted in a shorter duration of medication. This shift in focus may have contributed to the observed variations in medication duration in our study. 

Chanal et al. reported hospitalization of 6.6 ± 7.5 days (median 3.5, range 1–28) for patients with spontaneous LABD and 26 ± 25.5 days (median 18.5, range 6–90 days) for patients with drug-induced LABD [27]. Our research revealed an average of 1.3 ± 0.7 hospital visits and a length of hospitalization of 19.8 ± 19.7 days. Our study analyzed only dapsone as a treatment agent; therefore, we could not determine treatment regimens for other agents or the overall treatment regimen.

LABD is known from several case reports and reviews to be associated with systemic diseases such as UC, malignancy, and SLE. Genovese et al. reported four patients who had histories of neoplasm, two patients who were affected by UC, and one patient with coeliac disease as comorbidities in their study of 38 Italian patients [18]. Compared with an 0.12% estimated prevalence in the Italian general population, Genovese et al. reported a 5.6% prevalence of UC, which was much higher. 

There are several case reports and studies on the association between UC and LABD [8,28,29]. Shipman et al. reviewed 20 patients with UC and LABD and showed that UC occurred before LABD in all but two of the 20 cases [28]. Kanda et al. reviewed 33 reported cases of LABD associated with UC and showed that UC progressed with LABD in 94% of the patients [29]. In a study by Paige et al., 7.1% of 70 patients with LABD had UC, which is higher than the UK prevalence of UC (0.05%) [8]. In their study, UC developed before LABD in all patients, with a median of 6.5 years. The association between UC and LABD is unclear, but humoral- and cell-mediated immunity may be involved. The levels of pathogenic IgA autoantibodies in the sera of UC patients are thought to be higher than those in controls [8,30,31]. 

Tobon et al. [9] and Malipatel et al. [32] reported a case of linear IgA bullous dermatosis in a patient with SLE and suggested an association between LABD and SLE. 

LABD has been reported to be associated with malignancy. Among malignancies, the association between hematologic malignancies and LABD is more established than that with solid organ tumors. Andriano et al. reported a case of LABD in the setting of angioimmunoblastic T-cell lymphoma (AITL) and explained the pathophysiological process of dysregulation of somatic hypermutation and expression of chemokine receptor 5 in AITL, which leads to increased IgA [33]. Colmant et al. reported that a case of LABD associated with cutaneous involvement of an angioimmunoblastic T-cell lymphoma showed deep cutaneous involvement of the lymphoma with sub-epidermal blistering and direct immunofluorescence of heavy IgA linear deposits at the dermal–epidermal junction [34]. 

Waal et al. reported a case of LABD in a patient with renal cell carcinoma and suggested an association between LABD and malignancies [35]. Yang et al. reported a case of LABD in a patient with renal cell carcinoma and suggested a causal association between LABD and renal cell carcinoma based on remission after treatment for malignancy [36]. 

In our study, malignancy was the most commonly associated systemic disease, followed by SLE and UC. The time interval from malignancy diagnosis to LABD diagnosis was 1082 ± 974 days and the time interval from LABD diagnosis to malignancy diagnosis was 1188 ± 1160 days. The time interval from SLE diagnosis to LABD diagnosis was 527 ± 482 days, and the time interval from LABD diagnosis to SLE diagnosis was 1466 ± 1367 days. The time interval from UC diagnosis to LABD diagnosis was 1462 ± 1443 days, and the time interval from LABD diagnosis to UC diagnosis was 749 ± 1235 days.

Autoimmune blistering skin diseases, such as LABD, pose a challenge when attributing a specific drug as the cause. The most commonly reported causative drug is vancomycin [26]. Although LABD often develops in adults, there are few reports of drug-induced LABD in children. Children have been reported to develop LABD due to amoxicillin–clavulanate, trimethoprim–sulfamethoxazole, and nonsteroidal anti-inflammatory drugs [37,38,39].

More than 100 cases were induced by a wide range of drugs, especially antibiotics [17]. Baden et al. reported that vancomycin was the most frequent causative drug [40]. Subsequently, Chanal et al. demonstrated that vancomycin was the causative agent in 67% of cases [27]. Furthermore, Fortuna et al. reported that vancomycin was the most incriminated drug capable of inducing disease when single drugs were compared [24].

However, in our study, vancomycin use was infrequent, with only four individuals receiving it between 31 and 60 days before diagnosis, and six individuals receiving it within 30 days before diagnosis (Figure 5, Table S1). In our study, cephalosporin use was prominent, particularly the third (40 patients) and first generations (36 patients). 

Because of the rarity of LABD, conducting well-designed control studies is difficult, making it difficult to definitively establish the triggering role of a drug [17]. Identifying the specific drug responsible for drug-induced LABD can be challenging because of similarities in clinical, histological, and immunological features. 

To establish an association with drug-induced LABD, a widely accepted causality assessment such as the Naranjo adverse drug reaction (ADR) score is crucial, as proposed in previous reports. Furthermore, the French causality method, which is widely used in France and Europe, allows a clear distinction to be drawn between intrinsic imputability based on individual case characteristics and extrinsic imputability based on published data for each drug [17,41]. This method assumes equal or higher intrinsic imputability scores for all drugs regardless of their extrinsic imputability scores. Gentamicin and vancomycin were administered equally in this regard.

However, the reason for the differences between our study and previous studies remains unclear. Our study has a limitation in its design, as we were only able to identify the medications used by patients with LABD. However, we could not establish a causal relationship between the use of these medications and patient outcomes.

To the best of our knowledge, this is the first nationwide study on LABD. However, our study has several limitations that must be acknowledged. First, the data collected from the HIRA database did not include patients’ clinical records. We were unable to identify a specific causative agent owing to database limitations. Second, the association between the prevalence of diseases, antibiotics, and the incidence of LABD may not be causally related and may have been a chance finding. Third, there were a number of limitations owing to the lack of diagnostic codes for LABD in the ICD and KCD. We included patients who were treated with dapsone and underwent biopsy within 6 months, based on our selection criteria for case definition. Therefore, we excluded patients who were not treated with dapsone, such as those who were treated with steroids only, off-label drugs, or drugs not covered by national insurance, or whose illness was self-limited. During dapsone shortages, identifying patients is difficult. We also missed patients who were diagnosed without biopsy. Because we did not consider combination treatments and focused only on dapsone, our study included shorter durations of medication.

## 5. Conclusions

This is the first nationwide study of LABD in Korea to have evaluated patients from 1 January 2010 to 31 July 2022. Our study showed an annual incidence of LABD of 1.3 per 100,000 persons and a mean age at diagnosis of 55.9 ± 21.7 years. We also found the mean duration of dapsone treatment to be 30.7 ± 56.7 days. The proportions of patients with malignancy, SLE, and UC before and after the diagnosis of LABD were 16.9%, 2.3%, and 1.3%, respectively. The antibiotics used before LABD diagnosis were third-generation cephalosporins, followed by first-generation cephalosporins and ureidopenicillins. The clarity of diagnostic criteria and diagnostic codes will help improve research on LABD. Future study should investigate the causes of different rates of incidence between this study and other studies.

## Figures and Tables

**Table 1:** Characteristics of Patients.

Variables		N (%)	Annual Incidence *
Total number of patients		670 (100.0)	1.3
Mean age (years)		55.9 ± 21.2	
Age group			
	0–19 years	46 (6.9)	0.5
	20–39 years	105 (15.7)	0.7
	40–59 years	199 (29.7)	0.5
	≥60 years	320 (47.8)	3.2
Sex			
	Male	394 (46.2)	1.2
	Female	458 (53.8)	1.4
Location			
	Seoul	269 (23.1)	2.7
	Busan	14 (6.4)	0.4
	Incheon	53 (5.1)	2.1
	Daegu	22 (5.6)	0.7
	Gwangju	82 (4.4)	5.6
	Daejeon	27 (4.4)	1.8
	Ulsan	29 (1.5)	2.5
	Gyeonggi	71 (22.8)	0.6
	Gangwon	36 (3.6)	2.3
	Chungbuk	-	0.0
	Chungnam	-	0.0
	Jeonbuk	47 (3.5)	2.5
	Jeonnam	2 (2.3)	0.1
	Gyeongbuk	15 (3.1)	0.6
	Gyeongnam	-	0.0
	Jeju	3 (1.8)	0.5
	Sejong		0.0
Insurance type			
	Medical insurance	610 (91.0)	1.2
	Medical aid	56 (8.4)	0.1
	Free	4 (0.6)	0
Clinical course			
	Dapsone medication (days)	30.7 ± 56.7	
	Hospital visit (days)	1.3 ± 0.7	
	Hospitalization (days)	19.8 ± 19.7	

* All rates are per 100,000 people, directly age-adjusted to the 2021 population.

**Table 2:** LABD-associated systemic diseases.

Disease		Total N (%)	Before Diagnosis of LABD	After Diagnosis of LABD		
				Within 6 Months	After 6 Months	Total
Ulcerative colitis						
	Number of patients	9 (1.3)	4	3	2	5
	Time interval to diagnosis (days)		1462 ± 1443	72 ± 48	1764 ± 1631	749 ± 1235
Systemic lupus erythematosus						
	Number of patients	16 (2.3)	6	2	8	10
	Time interval to diagnosis (days)		527 ± 482	27 ± 9	1826 ± 1289	1466 ± 1367
Malignancy						
	Number of patients	113 (16.9)	40	21	52	73
	Time interval to diagnosis (days)		1082 ± 974	60 ± 60	1644 ± 1078	1188 ± 1160

## Data Availability

The data presented in this study are available on request from the corresponding author.

## Supplementary Materials

The following supporting information can be downloaded at: https://www.mdpi.com/article/10.3390/jcm13041159/s1, Table S1. Frequency of antibiotic use concurrent with LABD biopsy.
