# Supplementary material for: Linear IgA Bullous Dermatosis in Korea Using the Nationwide Health Insurance Database
Source: J Clin Med. 2024 Feb 19;13(4):1159. doi: 10.3390/jcm13041159 (PMC10889246; doi:10.3390/jcm13041159)
Supplement: Supplementary file 1 [file jcm-13-01159-s001.zip › jcm-2858965-supplementary.pdf]

Table S1. Frequency of antibiotic use concurrent with LABD biopsy

	Before skin biopsy		
	31–60 days	within 30 days	Total
Vancomycin	4	6	10 (1.5)
Quinolones	5	6	11 (1.6)
4th-generation cephalosporin	2	4	6 (0.9)
3rd-generation cephalosporin	14	26	40 (6.0)
2nd-generation cephalosporin	5	7	12 (1.8)
1st-generation cephalosporin	13	23	36 (5.4)
Penicillin	0	0	0 (0)
Carboxypenicillin	0	0	0 (0)
Ureidopenicillins	9	9	18 (2.7)
Aminopenicillins	3	9	12 (1.8)
Monobactam	0	0	0 (0)
Oxazolidinone	0	0	0 (0)
Anti-tuberculosis	0	2	2 (0.3)
Anti-malarial	1	0	1 (0.1)
Carbapenem	4	7	11 (1.6)
Non-vancomycin glycopeptides	1	2	3 (0.4)
Cyclines	1	4	5 (0.7)
Antifolate	1	4	5 (0.7)
Dapsone	1	30	31 (4.6)
Polymixin	0	0	0 (0)
Aminoglycoside	1	4	5 (0.7)
Nitroimidazole derivatives	0	0	0 (0)
Fusidate	0	0	0 (0)
Macrolide	4	7	11 (1.6)
Lincosamides	1	1	2 (0.3)
Fosfomycin	0	0	0 (0)
Peptidyl transferase	0	0	0 (0)
